# Glioblastoma Multiforme: Novel Therapeutic Approaches

**DOI:** 10.5402/2012/642345

**Published:** 2012-02-08

**Authors:** Arsenio M. Fialho, Prabhakar Salunkhe, Sunil Manna, Sidharth Mahali, Ananda M. Chakrabarty

**Affiliations:** ^1^Instituto de Biotecnologia e Bioengenharia (IBB) and Departamento de Bioengenharia, Instituto Superior Técnico, 1049-001 Lisbon, Portugal; ^2^Amrita Therapeutics Ltd., S. G. Highway, Thaltej, Ahmedabad 380 054, India; ^3^Laboratory of Immunology, Centre for DNA Fingerprinting and Diagnostics, Nampally, Hyderabad 500 001, India; ^4^Department of Microbiology and Immunology, University of Illinois College of Medicine, 835 South Wolcott Avenue, Chicago, IL 60612, USA

## Abstract

The current therapy for glioblastoma multiforme involves total surgical resection followed by combination of radiation therapy and temozolomide. Unfortunately, the efficacy for such current therapy is limited, and newer approaches are sorely needed to treat this deadly disease. We have recently described the isolation of bacterial proteins and peptides with anticancer activity. In phase I human clinical trials, one such peptide, p28, derived from a bacterial protein azurin, showed partial and complete regression of tumors in several patients among 15 advanced-stage cancer patients with refractory metastatic tumors where the tumors were no longer responsive to current conventional drugs. An azurin-like protein called Laz derived from *Neisseria meningitides* demonstrates efficient entry and high cytotoxicity towards glioblastoma cells. Laz differs from azurin in having an additional 39-amino-acid peptide called an H.8 epitope, which allows entry and high cytotoxicity towards glioblastoma cells. Since p28 has been shown to have very little toxicity and high anti-tumor activity in advanced-stage cancer patients, it will be worthwhile to explore the use of H.8-p28, H.8-azurin, and Laz in toxicity studies and glioblastoma therapy in preclinical and human clinical trials.

## 1. The Blood-Brain Barrier: A Major Barrier to Glioblastoma Therapy

The brain is a protected organ and therefore limits the number of compounds that can enter it from peripheral circulating blood. Thus only selected compounds such as glucose, alcohol, nicotine, and others can enter the brain to help nourish it or affect it in other ways, but many other blood components cannot enter the brain. This physical barrier, termed the blood-brain barrier (BBB), is characterized by monolayers of endothelial cells that are tightly packed to prevent leakage of brain components or entry of nonpermissive substances [1]. This entry is usually confined to small molecules of about 500 to 600 daltons (Da) although delta sleep-inducing peptide or enkephalin analogs of higher molecular weights are also known to cross the BBB in sufficient amounts to affect brain function. To complement this BBB, there are also efflux proteins such as the P-glycoprotein that can pump out many compounds from the brain vasculature, adding to the restrictive entry to the brain.

The presence of the BBB, and the efficient efflux mechanism, has significantly limited the number of drugs that can enter the brain to treat brain pathologies, including brain tumors. Transmembrane diffusion, usually by small lipophilic molecules, has been the major route to designing drugs that can enter the brain in amounts sufficient to provide effective treatment, although such molecules can be targets of P-glycoprotein efflux [2]. Since most of the compounds that can penetrate the BBB either employ various transporters or even transmembrane diffusion, current drugs are designed with the aim to use such mechanisms, as well as to interfere with the efflux mechanism of the P-glycoproteins.

## 2. Designing Drugs to Cross the BBB

The entry limitations to the brain of most drugs intended for the treatment of brain pathologies have triggered a flurry of activities to design drugs, lipophilic compounds with polar groups, for example, that can cross the BBB to enter the brain parenchyma by disrupting the tight junctions of the endothelial cells of the brain capillaries. One approach uses convection-enhanced delivery of the drugs through insertion of selected catheters containing drugs that penetrate the interstitial space to enter the brain parenchyma. Another development involves the use of recombinant adeno-associated virus expressing neurotrophic factors [3]. Physical disruption of the BBB can also be achieved through high localized osmolarity in the intracarotid administration of hypertonic mannitol solution followed by appropriate drugs [4]. The most active area of neuroactive drug development involves the use of transporters present on the capillaries of the neurons that normally transport essential nutrients to the brain through transcytosis. Such receptors, for example, insulin receptor, transferrin receptor, low-density lipoprotein (LDL) receptor, and so forth, can then endocytose any drug or foreign protein conjugated to it, from the luminal side to the abluminal side of the brain capillary endothelium [5] including the use of receptors for LDL (LRP) which are often overexpressed in tumors such as glioblastomas [6]. Targeted delivery of proteins, such as lysosomal enzymes or green fluorescent protein, to the neurons and astrocytes through use of lentivirus vector system and LRP-binding domain of apolipoprotein B fused to the respective protein, has shown great promise in potential therapeutic applications for central nervous system (CNS) disorders [7]. Similar viral vectors have been used for potential glioblastoma gene therapy through delivery of various genes that promote cell death [8].

The transcytosis of a carrier protein through binding with the ApoB LDL receptor binding domain of about 38 amino acids has prompted investigations to use a family of peptides derived from proteins that utilize LRPs for crossing the BBB to deliver therapeutic agents [9, 10]. An example of such peptides would be Angiopeps, a family of 19 amino acid long peptides that are efficient in using LRP type 1 to enter the brain. One such peptide Angiopep-2 has been conjugated with paclitaxel to give rise to ANG1005. The efficient transcytosis of ANG1005, as compared to paclitaxel alone, and its anticancer activity *in vivo*, has been demonstrated by enhanced survival of mice with implanted tumor [10]. Phase I human clinical data show encouraging efficacy of ANG1005 against recurrent malignant gliomas, as reported so far [11]. Other peptides such as TAT, homeodomain of Antennapedia, and SynB have also been used, conjugated to therapeutic compounds and with or without exposure on the surface of nanoparticles, for entry into the neurons across the BBB [12].

## 3. Current Trends in Glioblastoma Therapy

Glioblastoma multiforme (GBM) is one of the most deadly gliomas because of its high genetic diversity, a complex vasculature giving rise to intratumoral pressure and invasiveness, and the lack of access of most drugs because of the presence of entry barriers including the BBB. The invasive nature of GBM, allowing it to spread throughout the CNS, makes the therapy all the more difficult, and the prognosis remains poor [13]. The current standard of care involves gross total surgical resection followed by combination of radiotherapy with temozolomide and the continued adjuvant temozolomide therapy. The radiation therapy for GBM involves focal, fractionated external beam therapy while current drugs are alkylating agents such as temozolomide, often in combination with inhibitors of growth factors that promote tumor growth such as inhibitor (erlotinib) of epidermal growth factor (EGF) receptor [14]. Even with such combination therapy, the survival benefits have been minimal, demonstrating a need for improved therapeutic intervention. To overcome the drug entry problem, attempts are being made to use devices that can directly deliver a drug to the tumor. A drug- (carmustine-) impregnated wafer can be placed in the cavity after tumor resection or other anticancer agents can be used in drug-eluting catheters placed in the cavity after resection. The failure of most agents that hit a single target, including monoclonal antibodies such as Avastin that target the vascular endothelial growth factor (VEGF) and other antiangiogenic agents [15], eliciting quick drug resistance as well as giving rise to significant toxicity, clearly requires a new approach and a new conceptual framework for the treatment of cancers in general and GBM in particular [16, 17].

## 4. Bacterial Proteins and Peptides in Potential GBM Therapy: Novel Approaches and New Horizons

As mentioned above, only selected molecules such as glucose, insulin, and ethanol can cross the BBB to reach the brain parenchyma and many blood components cannot cross this barrier, yet certain extracellular bacterial pathogens that are basically normal inhabitants in the pharyngeal region (*Neisseria meningitides*, *Streptococcus pneumoniae*, etc.) or in the gastrointestinal tract (*Escherichia coli* strain K1) can cross the BBB to cause disease without having to hide in leukocytes for crossing the BBB [18–20]. Although the transcytosis of such bacteria through human brain microvascular endothelial cells has been well established [21], very little is known about how such a bacterium, *N. meningitides*, for example, actually crosses the BBB to invade the brain meninges. Certain bacterial components, the capsules, and virulence factors such as factor H binding protein or iron acquisition system, as well as pili such as type IV pili, have been implicated in the successful adhesion, host signaling to disrupt the BBB, and binding to the meninges [21]. No single bacterial component has been shown to allow *N. meningitides* for efficient transcytosis to the brain meninges.

An important aspect of basic research is the unpredictability of the direction it will take. Thus fundamental basic research has contributed to many out-of-the-box ideas and new approaches for drug development and therapeutic intervention. Let us give an example. About 12 years ago, we had no interest or expertise in cancer research, including development of anticancer drugs. We were studying the mechanism of infection of a bacterium known as *Pseudomonas aeruginosa* in the lungs of cystic fibrosis (CF) patients. Similar to the long-term nasopharyngeal colonization by *Neisseria meningitides*, *Streptococcus pneumonia*, and others mentioned earlier, chronic infections in the lungs of CF patients are known to be caused by *P. aeruginosa* by forming a biofilm on the epithelial cells of the lung through production of various polysaccharides. The slow-growing biofilms take up little nutrients and produce little toxic metabolites in the host, but are rendered resistant to antibiotics and immune attack and are hard to eradicate. Since *P. aeruginosa* is an opportunistic pathogen that normally does not infect healthy people with normal immune function, and CF patients have normal immunity, we were interested in knowing if the infecting *P. aeruginosa* cells produce any new toxin in the environment of the CF lung that might be cytotoxic for the foot soldiers of the immune system such as neutrophils and macrophages. We, therefore, tested the growth media of a clinical isolate of *P. aeruginosa* from the sputum of CF patients for cytotoxicity against J774 cells, which are widely used as a cell line with macrophage activity. Indeed, the growth medium showed the presence of high cytotoxic activity against such J774 cells. Further fractionation of the growth medium showed the presence of two proteins, azurin and cytochrome c551, which retained the cytotoxic activity against J774 cells. Azurin, in particular, showed strong cytotoxic activity, although azurin was believed at that time to be involved in electron transport in *P. aeruginosa* as a redox protein with cytochrome c551 as its electron transfer partner.

Highly encouraged by these initial observations that we have found a new toxin against macrophages (and a new hitherto unknown function for azurin), our group checked the cytotoxic activity of purified azurin against primary mouse macrophages isolated from the peritoneum. To our utter surprise, azurin again demonstrated high cytotoxicity in J774 cells but very little cytotoxicity for mouse primary peritoneal macrophages. Repeated experiments showed the same results. With a great deal of confusion and anguish, we searched for the difference between J774 cells and normal peritoneal macrophages, until it dawned to us that J774 macrophage cell line is derived from tumors, allowing them to grow rapidly in cell culture and facilitating their use as macrophages.

Does azurin demonstrate cytotoxicity against tumor cells but not against normal cells like peritoneal macrophages? Further experiments clearly demonstrated that azurin not only had high cytotoxicity against human cancer cell lines such as melanoma and breast cancer showing very little toxicity in normal cells, but also showed a great deal of promiscuity in attacking and demonstrating high cytotoxicity against viruses such as the AIDS virus HIV-1 or parasites such as the malarial parasite *Plasmodium falciparum* or the toxoplasmosis-causing parasite *Toxoplasma gondii* [22, 23]. Such promiscuity of azurin was not only confined to its anticancer, antiviral; or antiparasitic activities, but extended to multiple steps in cancer growth progression pathways that were significantly inhibited by azurin. For example, azurin enters preferentially to cancer cells than to normal cells and inhibits the cancer cell growth by interfering in multiple steps in its growth such as receptor tyrosine-kinase-mediated cell signaling, EGF-EGFR-mediated angiogenesis and inducing apoptosis through stabilization of tumor suppressor p53 by preventing its ubiquitination. No rationally designed drug can match this kind of mode of anticancer action (interference in multiple steps) of azurin, including its entry specificity in cancer cells [23]. It should be emphasized that these were the pathways that we studied with respect to cancer growth inhibition by azurin. It is likely that azurin may impact many other pathways in cancer cell growth that only an intelligently designed weapon produced by bacteria with 3 billion years of evolutionary wisdom can target to keep cancers in check. Because such a bacterial protein weapon that forms complexes with many human/eukaryotic proteins important for disease progression must work in an animal or human cellular environment where the bacteria reside, it is interesting to note that azurin structurally resembles immunoglobulins, making it mostly a nonimmunogenic protein, so that it would not be eliminated by the body's immune system when released from bacteria to attack cancer cells.

### 4.1. Azurin-Like Protein, Neisseria meningitides, and Glioblastoma

**Figure 1:**
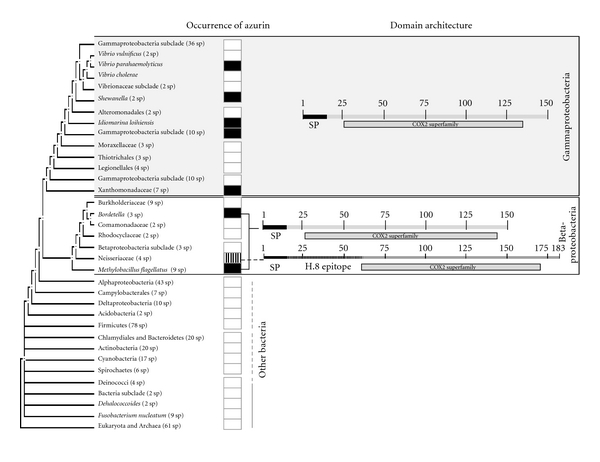
Occurrence of azurin-like proteins across a phylogenetic tree. By homology searches, azurin orthologous were found in a variety of bacterial species members of the gamma- and betaproteobacteria but absent from the other bacterial phyla and the Eukarya. Black and white boxes indicate presence and absence of azurin, respectively. The box with vertical bars indicates the existence of an azurin-like protein (termed Laz) in *Neisseria* species. Shown on the right are the schematic representations of the conserved core domain presented in azurin-like proteins (COX2 superfamily). The phylogenetic tree was drawn using STRING [26]. SP: signal peptide.

Azurin, 128 amino acid long and about 14 kDa, is not only produced by *P. aeruginosa* but by many other bacteria. Azurins are found in some members of the gamma and beta subdivisions of the Proteobacteria but absent from the other bacterial phyla and the Eukarya (Figure 1). They are members of the cupredoxin superfamily represented by water-soluble small copper-containing proteins (10–20 kDa) involved in electron transfer reactions [24, 25]. Although the sequence homology between the azurins varies between 50 and 90%, the structural homology between these molecules is highly conserved. All azurins have a characteristic single-domain signature that consists of a compact structurally rigid *β*-sandwich core (immunoglobulin fold) formed by two main *β*-sheets made up of seven or more parallel and antiparallel strands (Greek key *β*-barrel structure). In addition, azurins possess an essentially neutral hydrophobic patch surrounding the copper site [25].

**Figure 2:**
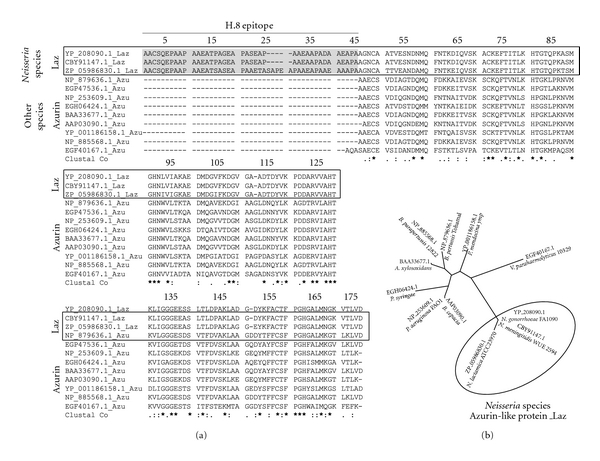
(a) A multiple amino acid alignment of 9 representative bacterial azurins and 3 azurin-like proteins (Laz) from neisserial species. Identical residues (*⋆*), conservative amino acid substitutions (:), and semiconservative amino acid substitutions (.) are shown below the aligned sequences. Laz and azurins that were used included are the following: Laz from *N. gonorrhoeae* FA 1090 (YP_208090.1); H.8 outer-membrane lipoprotein from *N. meningitidis* WUE 2594 (CBY91147.1); azurin from *N. lactamica* ATCC 23970 (ZP_05986830.1); azurin from *Bordetella pertussis* Tohama I (NP_879636.1); azurin from *Achromobacter xylosoxidans* AXX-A (EGP47536.1); azurin from *P. aeruginosa* PAO1 (NP_253609.1); azurin from *Pseudomonas syringae* pv. glycinea str. race 4 (EGH06424.1); azurin from *Achromobacter xylosoxidans* (BAA33677.1); azurin from *Burkholderia cepacia* (AAP03090.1); azurin from *Pseudomonas mendocina* ymp (YP_001186158.1); azurin from *Bordetella parapertussis* 12822 (NP_885568.1) and azurin from *Vibrio parahaemolyticus* 10329 (EGF40167.1). The CLUSTAL X software [29] was used to generate this multiple sequence alignment. (b) A phylogenetic tree calculated from the alignment represented above in (a). Clustal X with neighbour-joining method [30] was used to construct the tree.

 Of interest is the presence of an azurin-like protein, termed Laz, uniquely found in *Neisseria* species including the *meningococcus* and the *gonococcus* (Figure 1). Unlike other bacterial azurins where azurin is periplasmic, *Neisserial* azurin is surface exposed and harbors an additional 39 amino acid moiety in its N-terminal called an H.8 epitope [27, 28]. Beyond this 39 amino acid N-terminal region in Laz is a 127-amino-acid azurin domain at the C-terminal that is highly homologous to the *P. aeruginosa* azurin (Figure 2). The size of the Laz protein can be somewhat variable among the *Neisseria* species but generally contains imperfect AAEAP pentameric repeat motifs in the H.8 epitope, no aromatic amino acids, and a lipid-modified N-terminal cysteine residue (Figure 2) [28].

Azurin is believed to be a weapon produced by some pathogenic bacteria such as *P*. *aeruginosa* or *N. meningitides*, which reside in the human body and where azurin is released in response to invasion by internal or external invaders such as cancers, viruses, and parasites, which can cause harm to the host and causing loss of sanctuary for the bacteria [22, 23]. It is thus interesting to note that *Neisseria *such as *N. meningitides* chose to put an additional armor, such as the H.8 epitope, in its weapon azurin, giving rise to Laz. Why does *N. meningitides* need this additional armor that *P. aeruginosa* does not? A look at the residency pattern of these two opportunistic pathogens demonstrates that while *P. aeruginosa* can seldom cross the BBB to invade the brain meninges to reside there, *N. meningitides* is known to do so, as mentioned earlier. While resident in the brain meninges, *N. meningitides* may feel threatened to lose its habitat if a brain tumor, such as a glioblastoma, crops up in the vicinity. Similar to the BBB, the glioblastoma may have tight junctions that may prevent azurin to enter the tumor to kill it and azurin needs an additional component to help it disrupt such an entry barrier. Additionally, instead of being periplasmic as for all other azurins, surface exposure of Laz allows this armor (H.8 epitope) to help in the transcytosis of the bacteria to cross the BBB as well.

Does the presence of the H.8 epitope in azurin, that is, Laz, allow facilitated disruption of any entry barrier to the glioblastomas or even help in crossing the BBB? Even more pertinent is the question: does the bacterial weapon azurin work in the human environment to attack tumors and cause their regression?

Because azurin and Laz are bacterial proteins, for preclinical or human clinical trials, they face stringent regulation and may have toxic cellular contaminants. On the other hand, a peptide derived from azurin or Laz, which can be chemically synthesized as a drug, has entry specificity in cancer cells and demonstrated anticancer activity and therefore can be tested clinically for toxicity and efficacy in humans. Such a peptide is p28, a 28-amino-acid peptide derived from azurin (azurin 50–77) with entry specificity in cancer cells, as well as with anticancer activity [31]. Eleven US patents have been issued on these candidate drugs since 2006 (Table 1) covering the use of a single protein or peptide drug for multiple diverse diseases. A start-up company, CDG Therapeutics Inc. (CDGTI), sponsored both preclinical and human phase I clinical trials with p28 as an anticancer agent. In preclinical trials, p28 showed no toxicity or immunogenicity in animals, including monkeys, which prompted a phase I human clinical trial in Chicago. This trial comprised 15 advanced-stage cancer patients with metastatic, refractory solid tumors (7 melanoma, 4 colon, 2 sarcoma, 1 pancreatic, and 1 prostate) and with an average life expectancy of less than 6 months and where the tumors were no longer responding to conventional drugs. When chemically synthesized p28 was given as an intravenous bolus in 5 escalating doses for 4 weeks, followed by a 2-week break before the next higher dose, several patients showed partial regression of their tumors with 2 patients (1 melanoma, 1 sarcoma) showing complete regression. Very little toxicity was seen even with the highest concentration of p28, and 1 patient, out of 6 surviving patients, has been living disease-free for over 80 weeks ([32] and http://www.cdgti.com/). 

The fact that out of 15 patients where the tumors were refractory to all conventional drugs, 2 patients showed complete regression of their tumors with enhanced life expectancy raises important questions not only about the unique mode of action of p28, and therefore azurin, but also about the genetic traits, either in the tumor genome or in the patient genome, that make these tumors highly susceptible to p28. The complete tumor regression may also be due to the right concentrations of p28 and the right length of time of treatment. It should be noted that p28 is only a part of azurin, and there are other domains in azurin, p26, for example, that demonstrate anticancer activity through inhibition of receptor tyrosine kinases such as EphB2 [33], or others. Thus azurin will likely be a more potent anticancer agent than p28, provided its lack of toxicity and immunogenicity in humans can be demonstrated.

**Figure 3:**
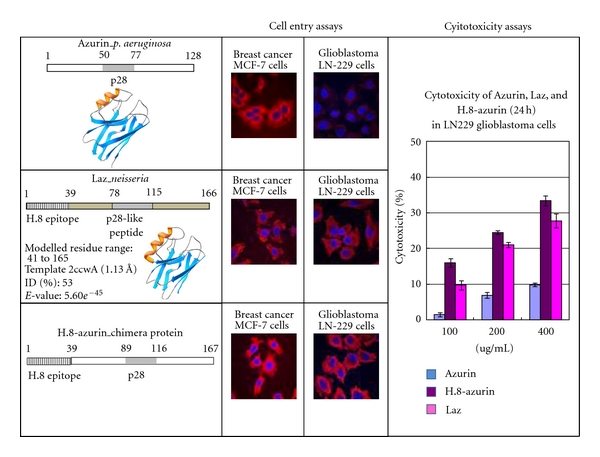
Left row: structural depiction of azurin, Laz (with the H.8 epitope in the N-terminal), and azurin with the cloned H.8 epitope in its N-terminal. The area corresponding to the p28 peptide region is marked. Middle row: the entry of fluorescently labeled azurin, Laz, and H.8-azurin in breast cancer MCF-7 and glioblastoma LN-229 cells is shown. The red color reflects the Alexa-Fluor-568-conjugated protein while the blue color represents the nucleus stained with DAPI. Right row: cytotoxicity of azurin, H.8-azurin, and Laz at 3 different concentrations towards LN-229 glioblastoma cells. The detailed methodologies have been described by [28].

The presence of an additional H.8 epitope in neisserial azurin (Laz) begs the question if indeed the H.8 epitope facilitates the anticancer weapon azurin to cross any entry barrier to glioblastomas, and given the surface exposure of Laz in *N. meningitides*, whether the H.8 epitope might even be involved in facilitated BBB crossing that *N. meningitides* must accomplish to invade the brain meninges. We have, therefore, tested the entry specificity of *P. aeruginosa* azurin (Paz), neisserial azurin (Laz), and an additional construct H.8-azurin where the H.8 epitope from Laz was cloned in the N-terminal part of Paz to produce the chimeric protein H.8-azurin (Figure 3). The entry specificity and cytotoxicity of these three proteins were then tested in human breast cancer MCF-7 and glioblastoma LN-229 cells [28, 34]. As shown in Figure 3 (top row), when azurin was conjugated to a fluorescent red dye Alexa Fluor 568 and looked for entry in breast cancer MCF-7 as well as the glioblastoma LN-229 cells, it entered the MCF-7 cells very efficiently but the entry to LN-229 cells was limited and inefficient. In contrast, Laz, with the H.8 epitope in its N-terminal, entered both MCF-7 and LN-229 cells efficiently (Figure 3, middle row). Most interestingly, when the H.8 epitope was introduced in the N-terminal part of *P. aeruginosa* azurin, this chimeric protein could enter both MCF-7 and LN-229 cells very efficiently, similar to Laz but very unlike *P. aeruginosa* azurin (Figure 3, bottom row). This clearly suggests that the H.8 epitope significantly facilitates the entry of azurin to glioblastoma cells.

How may the H.8 peptide facilitate such entry of azurin in glioblastoma cells? Does it disrupt any entry barrier, perhaps the tight junctions in the LN-229 cells? To address such questions, we used chemically synthesized 39-amino-acid H.8 peptide by itself, labeled with Alexa Fluor 568, for its entry or unlabeled H.8 peptide for cytotoxicity in LN-229 and MCF-7 cells. The H.8 peptide by itself did not show any entry or cytotoxicity in these cancer cells [28]. While azurin by itself showed much less cytotoxicity in glioblastoma LN-229 cells as compared to H.8-azurin and Laz (Figure 3, cytotoxicity assays), when the chemically synthesized H.8 epitope peptide was present along with azurin as a mixture (as compared to H.8-azurin where it was fused to azurin), the mixture showed a much higher entry level of azurin, and corresponding cytotoxicity, than azurin alone, clearly demonstrating a role of the H.8 epitope in facilitating the entry of azurin, or perhaps any other drug, to the glioblastoma cells [28].

**Figure 4:**
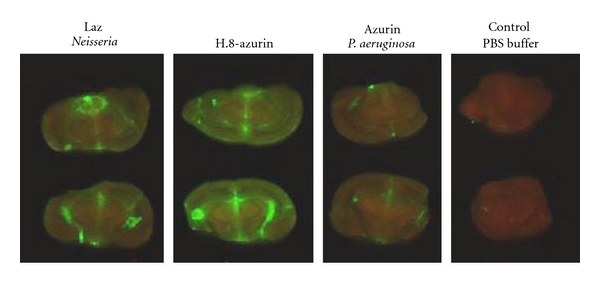
Odyssey scanning of brains from mice previously injected peritoneally with green fluorescent IR-dye-conjugated Laz, H.8 azurin, and azurin (from *P. aeruginosa*). Individual mice were injected with individual fluorescent protein, and the mice were sacrificed after 24 hours to obtain the brains for Odyssey imaging.

Does the H.8 epitope promote the crossing of the BBB as well? Azurin, H.8-azurin, and Laz were each fluorescently labeled with green fluorescing IR dye 800 CW (LI-COR Biotechnology, Lincoln, Nebraska) and 500 *μ*g of each was injected intraperitoneally in live nude mice to look for their transport to the brain. After 24 hours, the mice were sacrificed, the brains isolated, and the fluorescent images were measured with a LI-COR Odyssey Infrared Imaging System. Very little fluorescent azurin was detected in the brain, while both fluorescent Laz, and particularly H.8-azurin, were detected at a much higher level (Figure 4), suggesting that the presence of the H.8 epitope in azurin allowed this protein to more efficiently cross the BBB to reach the brain than free azurin [34].

## 5. Concluding Remarks

Surgery, radiation, and chemotherapy, the main approaches to cancer therapy, do not work very well for glioblastoma mainly for two reasons. Firstly, few chemotherapeutic drugs can cross the BBB to reach the brain tumor in significant amounts for therapeutic purposes. A second problem involves the high invasive nature of glioblastomas within the central nervous system, greatly accelerating the chances for relapse of the tumor. Thus any approach to glioblastoma therapy must take into consideration not only the need for complete tumor removal but also to reduce the possibility of a relapse. It is interesting to point out that azurin has both these properties. Azurin and p28 not only have anticancer activity, interfering in multiple steps in cancer growth, but also have cancer preventive properties, as determined by inhibition of the oncogenic transformation of normal mouse mammary cells to develop precancerous lesions in presence of a potent carcinogen, 7,12-dimethyl-benz-anthracene [35]. The lack of toxicity of p28 in advanced-state cancer patients has been demonstrated, as stated earlier. It is likely that azurin, and hopefully Laz and H.8-azurin, will prove to be nontoxic in human. Although their stability in serum is likely going to be low, not only Laz or H.8-azurin can potentially be effective in postsurgical glioblastoma patients but also perhaps these two proteins, and azurin and p28, can be effective in preventing the emergence of tumors in women with BRCA1/BRCA2 mutations. These mutations in the human genome are known to make women (and to some extent men) susceptible to breast and ovarian (and in case of men, prostate) cancers. Once the genetic tests show the presence of such mutations in a person with a family history of breast cancer, that person has very little recourse other than surgery even before the cancer appears. As technologies advance, and solid state peptide synthesis extends to peptides of 100 to 150 amino acid length as it is now, it might be possible to chemically synthesize azurin, H.8-azurin, or Laz as potential drugs, similar to p28. The only other issue would be to conjugate them with compounds that would make them suitable to be taken orally rather than through intravenous injections. That will make p28, azurin, H.8-azurin or Laz “a pill a day to keep cancers away.” Alternatively, given azurin's structural similarity to immunoglobulins, lack of its immunogenicity, and possible lack of toxicity, it should be possible to use gene therapy [7, 8] to allow its expression from the human genome, thus guarding the human body from invasion not only by cancers, but also by viruses such as HIV-1 or parasites such as the malarial parasite [23].

**Figure 5:**
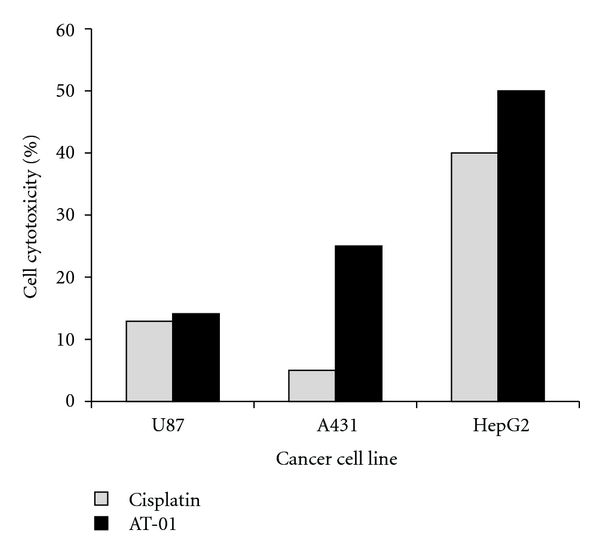
Cytotoxicity effect of AT-01 peptide and cisplatin at 1 *μ*M on U87 human glioblastoma, A431 skin cancer, and HepG2 liver cancer cell lines. Cancer cells (1 × 10^4^) were seeded, and after 24 hours the cells were treated separately with 1 *μ*M concentration of AT-01 peptide and cisplatin as a positive control. The viability of the cells was estimated by using MTT assay on the basis of formazan formed, which was detected spectrophotometrically by measuring optical density at 595 nm at 24 hours, and % cell cytotoxicity was determined [28].

Finally, the efficacy of p28 in allowing complete regression of tumors in a melanoma and a sarcoma patient, where the tumors were nonresponsive to conventional drugs, raises some interesting questions. Apart from the question of the genotypic characteristics of the tumor or patient genomes as mentioned earlier, could it be due to the unique characteristics of the bacterial protein/peptide drugs that work through novel multiple pathways? It should be noted that p28, and in fact azurin, are known to enter breast cancer tumors very efficiently and allow significant tumor shrinkage *in vivo* in nude mice (reviewed in [23]). Unfortunately, no breast cancer patient was recruited for the phase I trial with p28 but subsequent phase II trials, hopefully recruiting newly diagnosed breast and other cancer patients, should shed considerable light on the scope of the efficacy of p28 for other cancers as well. The other important question is if azurin (p28) is a unique potential bacterial protein (peptide) anticancer drug or if other bacteria with long-term residence in the human body may produce similar protein weapons to defend their habitat (human body) from invaders like cancers, thus enlarging our repertoire of bacterial anticancer drugs. It is interesting to note that an Indian company, Amrita Therapeutics (http://www.amritatherapeutics.com/), has reported the isolation of a similar protein, ATP-01 (and a chemically synthesized peptide AT-01 derived from ATP-01) from other bacteria as well. Although completely different from azurin in amino acid sequence and other features, ATP-01 shows structural similarity to the immunoglobulin fold of azurin, a similar size (about 17 kDa) and with anticancer and anti-HIV/AIDS activity. The data in Figure 5 demonstrate the anticancer activity of the peptide AT-01 derived from the mycobacterial protein ATP-01. Although the cytotoxic activity of AT-01 is lower in glioblastoma cell line U87 than for skin or liver cancer cell lines, it is comparable to cisplatin at similar concentration, and perhaps this activity can be enhanced by fusing AT-01 with the H.8 epitope. Whether such bacterial protein/peptide drugs as azurin and ATP-01 or their peptides p28 and AT-01 will prove to be nontoxic and effective in cancer patients will determine if our next-generation anticancer drugs will be of microbial origin, perhaps rivaling the antibiotic industry.

## Figures and Tables

**Table 1:** List of issued US patents on protein drugs azurin and Laz.

Title	Inventors	Patent number	Date of issuance
Cytotoxic factors for modulating cell death	Chakrabarty AM, Das Gupta TK, Punj V, Zaborina O	7,084,105	August 1, 2006
Compositions and methods for treating HIV infection with cupredoxins and cytochrome c	Chakrabarty AM, Das Gupta TK, Yamada T, Chaudhari A, Fialho A, Hong CS	7,301,010	November 27, 2007
Compositions and methods for treating malaria with cupredoxin and cytochrome	Chakrabarty AM, Das Gupta TK, Yamada T, Chaudhari A, Fialho A, Hong CS	7,338,766	March 04, 2008
Compositions and methods for treating conditions related to ephrin signaling with cupredoxins	Chakrabarty AM, Das Gupta T, Yamada T, Chaudhari A, Fialho A, Zhu Y	7,381,701	June 03, 2008
Cytotoxic factors for modulating cell death	Chakrabarty AM, Das Gupta TK, Punj V, Zaborina O, Hiraoka Y, Yamada T	7,491,394	February 17, 2009
Compositions and methods for treating HIV infection with cupredoxin and cytochrome c	Chakrabarty AM, Das Gupta, T, Yamada, T, Chaudhari A, Fialho A, Hong CS	7,511,117	March 31, 2009
Compositions and methods to control angiogenesis with cupredoxins	Mehta RR, Taylor BN, Yamada T, Beattie CW, Das Gupta TK, Chakrabarty AM	7,556,810	July 07, 2009
Compositions and methods to prevent cancer with cupredoxins	Das Gupta TK, Chakrabarty AM	7,618,939	November 17, 2009
Cupredoxin derived transport agents and methods of use thereof	Chakrabarty AM, Das Gupta T, Yamada T, Fialho A	7,691,383	April 06, 2010
Compositions and methods for treating malaria with cupredoxin and cytochrome	Chakrabarty AM, Das Gupta T, Yamada T, Chaudhari A, Fialho A, Hong CS	7,740,857	June 22, 2010
Transport agents for crossing the blood-brain barrier and into brain cancer cells, and methods of use thereof	Hong CS, Yamada T, Fialho A, Das Gupta TK, Chakrabarty AM	7,807,183	October 5, 2010
